# Nano-aggregates of furan-2-carbohydrazide derivatives displaying enhanced emission with a bathochromic shift†

**DOI:** 10.1039/c9ra07290j

**Published:** 2019-11-06

**Authors:** Ge Ding, Xinchao Wang, Xiujuan Li, Hongpan Liu, Lunxiang Wang, Na Liu, Fang Gao, Zhenqiang Wang

**Affiliations:** College of Materials and Chemical Engineering, Chongqing University of Arts and Sciences Chongqing China 402160 dingge1989cqu@126.com; College of Pharmacy, Heze University Heze Shandong Province China 274000 wxc198566@126.com; College of Chemistry and Chemical Engineering, Chongqing University Chongqing China 400044 fanggao1971@126.com; College of Chemistry, Chongqing Normal University Chongqing China 401331 772630581@qq.com

## Abstract

The non-fluorescent Schiff base compound C1 (*N*'-((4′-ethyl-3-hydroxy-[1,1′-biphenyl]-4-yl)methylene)furan-2-carbohydrazide) in organic solvent (*e.g.*, THF) was found to produce yellow-green fluorescence emission upon addition of H_2_O, and granular-shaped aggregates in a THF/H_2_O mixed solution formed and exhibited obvious aggregation-induced emission (AIE). Especially its keto fluorescence band intensified dramatically, while the enol emission band remained almost unchanged. Hence, a change in fluorescence from no emission of light to emission of bright yellow-green light under a UV lamp was observed with the naked eye. In contrast, the reference compound C2 (*N*'-((4′-ethyl-3-methoxy-[1,1′-biphenyl]-4-yl)methylene)furan-2-carbohydrazide) showed no intensified fluorescence emission under the same experimental conditions. These results indicated the significant role played by intramolecular H-bonding in the formation of the C1 aggregates and the AIE process.

## Abstract 2

C1 exhibited obvious AIE phenomena. A change from a lack of fluorescence emission to the emission of yellow-green light under a UV lamp was observed upon the inclusion of water in the solvent.

## Graphical abstract

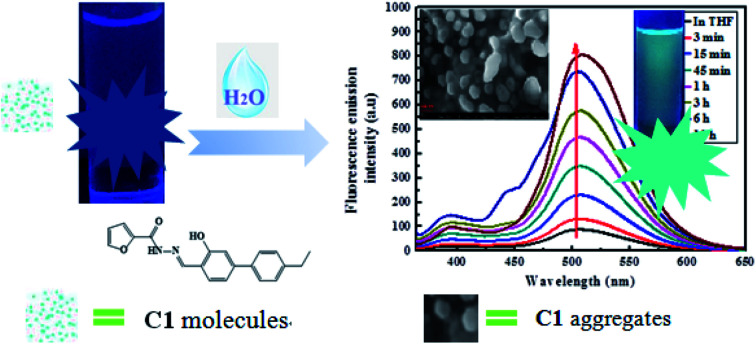

## Introduction

There has been considerable interest recently in fluorescent materials because of their various applications in organic light-emitting diodes (OLEDs), fluorescence-based sensors, and biological cell imaging. As an emerging class of fluorescent nanomaterials, carbon dots (CDs)^1–5^ and organic fluorescent nanomaterials have attracted sustained attention due to their superior luminescence, low toxicity, great stability, and ease of manufacture. Recently, dyes with aggregation-induced emission (AIE) properties have attracted intense research interest as a novel class of optical materials due to their molecular structures being much easier to modify and tune than the structures of CDs.^6,7^ AIE dyes are rotor-type molecules that have been reported to present weak fluorescence in pure organic solutions but strong fluorescence in the aggregated state.^8,9^ These observations have been attributed to the free motion of the rotor structures in solution consuming excited state energy by non-radiative decay to quench the emission – and the blocking of the intramolecular rotational motion and intramolecular vibration in the aggregated state activating the radiative decay pathway to yield strong fluorescence.^10,11^

Molecules that undergo excited-state intramolecular proton transfer (ESIPT) display a larger Stokes shift, which can effectively prevent self-absorption. Hence, a great deal of effort has been put into studying the optical properties of molecules displaying ESIPT with AIE.^12–14^ Several such molecules have been reported. The Yang group,^15^ for example, has obtained nanoscale homodispersed organic aggregates in poor solvents using the reprecipitation method and showed these aggregates displaying π–π stacking interactions.^16–18^ Similarly, the Li and Hou group^19^ has realized reversible color and fluorescence changes upon UV light irradiation with good fatigue resistance. The Mu group^20^ reported the use of AIE-displaying 2-(1-hydroxy-2-naphthyl)methylene hydrazine molecules for detecting metal ions, and realized simultaneously detection of Zn^2+^ and Co^2+^ in isolated and aggregated states, respectively. However, previous studies of the mechanism of the aggregation of molecules displaying ESIPT mainly focused on the π–π effect, which mainly blocks intramolecular rotational motion, but there have been few studies on the role of intramolecular H-bonding in the aggregation process.^21–23^

In this work, we synthesized a new conjugated chromophore carrying a proton transfer segment, and this chromophore produced enol and keto double emission bands. The intensity of the keto emission became dramatically higher during aggregation of the chromophore, while the enol emission remained almost unchanged. Hence, an intensified fluorescence of yellow-green light was observed with the naked eye during the aggregation process under UV lamp irradiation. A control experiment involving a reference molecule without an ESIPT segment was also conducted under the same experimental conditions, and showed no formation of aggregates in the mixture solution system and hardly any enhancement of the emission intensity. These results further indicated the important role played by intramolecular H-bonding in the formation of aggregates and the enhancement of the emission.

## Results and discussion

**Fig. 1:**
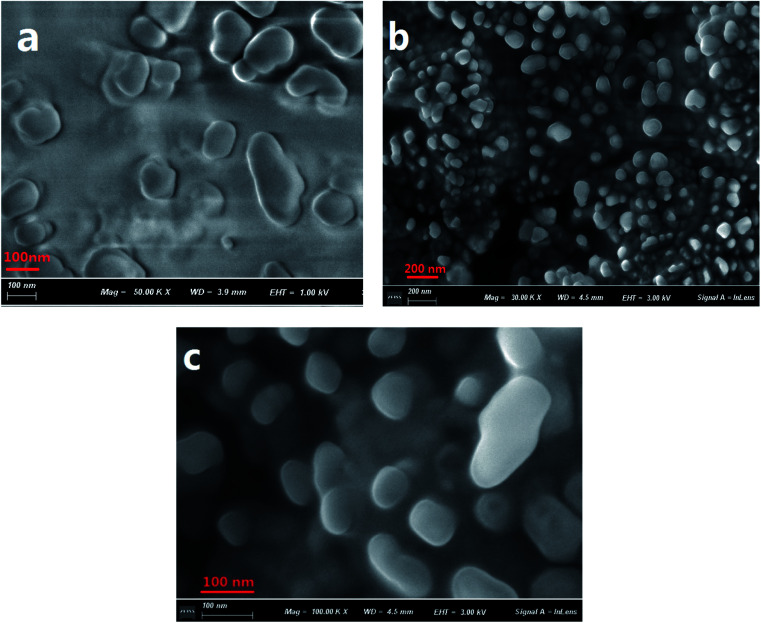
SEM images of C1 aggregate formation in a THF/H_2_O (40/60, v/v) solution (2 × 10^−5^ mol L^−1^) at 5 min (a) and 12 h (b and c). A magnified image is shown in (c).

The formation of aggregates of C1 in a THF/H_2_O (40/60, v/v) solution was monitored using scanning electron microscopy (SEM) (Fig. 1). During the first five minutes of aggregation, an amorphous state was observed. With increasing aggregation time (such as at 12 h), granular-shaped particles with dimensions of 100–200 nm were seen. However, under the same experimental conditions, the reference C2 in the THF/H_2_O mixed solution or in other mixed solution systems (such as EtOH/H_2_O, DMF/H_2_O) was not observed to form nano-aggregates. These results indicated a vital role for intramolecular H-bonding in the formation of aggregates of the target C1. An X-ray diffraction (XRD) pattern of powder C1 displayed intense diffraction peaks, while the reference C2 yielded no obvious diffraction peaks (Fig. S1, ESI†); these results suggested the formation of a crystalline state and a harmonious arrangement by C1 molecules in the solid state.

The sizes of the target dye C1 aggregates in the mixed THF/H_2_O solvent at various aggregation times were also measured using dynamic light scattering (DLS). The diameters of the aggregates in the mixed system were in the range 10–30 nm at an aggregation time of 30 min, and approximately 160–210 nm at 12 h (Fig. S2, ESI†). These results were in accordance with those obtained with the SEM images.

**Fig. 2:**
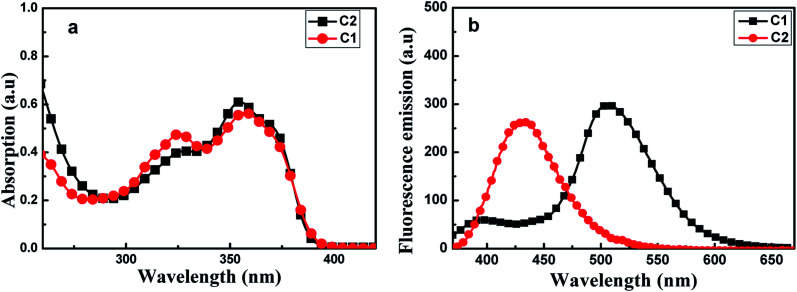
Absorption spectra (a) and fluorescence emission spectra (b) of C1 and C2, each at a concentration of 2 × 10^−5^ mol L^−1^ in THF. The excitation wavelength used was 350 nm.

Fig. 2(a) shows UV-visible absorption spectra of C1 and C2 in pure THF solutions. Each of these spectra showed two absorption peaks: one at ∼326 nm ascribed to (n, π) transition due to the presence of imino group; and the other at about 350 nm, which may have been produced by π, π transition due to intramolecular charge transfer. Furthermore, the absorption maxima of C1 were a bit red-shifted compared with those of C2.

As shown in Fig. 2(b), C1 yielded two emission bands in dilute THF with a small fluorescence quantum yield (*Φ*, 0.0219), while C2 yielded only a single emission band. For C1, the emission band at 370–425 nm was attributed to enol emission, while that at 475–550 nm was assigned to keto emission generated by an ESIPT reaction. This emission property proved that a proton transfer reaction can occur in C1, in its excited state, due to the presence of intramolecular H-bonding (Fig. S3, ESI†). The keto emission band of C1 showed a larger Stokes shift (∼152 nm), which could be attributed to energy loss during internal proton transfer in the excited state.

The optical properties of aggregates of C1 in various THF/H_2_O solutions with different volume ratios of THF to H_2_O were measured. For all such ratios tested, the absorption intensity of C1 decreased significantly with increasing aggregation time (Fig. S4(a), ESI†), but did so most significantly when the ratio was increased to 4 : 6, where it displayed a significant red shift (of about 6 nm) relative to the maximum absorption peak in pure THF. The decrease of absorption intensity may have been due to the continuous consumption of free molecules *via* formation of intermolecular π–π interactions. In contrast, the absorption spectra of C2 remained nearly unchanged at the same experimental conditions (Fig. S4(b), ESI†).

**Fig. 3:**
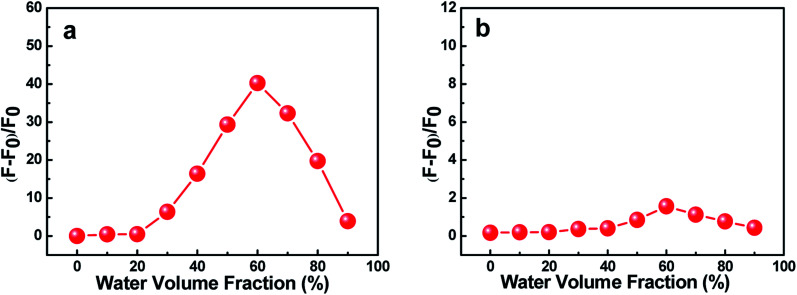
Effects of the water volume fraction (*f*_w_) of C1 (a) and C2 (b) on their fluorescence intensities in THF/H_2_O mixed solvents at 12 h. The concentration of C1 and that of C2 were both 2 × 10^−5^ mol L^−1^.

The effects of water volume fraction (*f*_w_) in the THF/H_2_O mixed solvent on the fluorescence intensities of C1 and C2 were also investigated (Fig. 3). The emission spectra of C1 for different ratios of THF to H_2_O (water fractions from 0 to 90%, v/v) were obtained at an aggregation time of 12 h (Fig. 3(a)). These spectra showed that the emission intensity was greatest, with a fluorescence quantum yield of 0.435 (19.86 times than that in pure THF solution), when the water fraction in the THF/H_2_O mixed solution was 60%, which further indicated C1 in THF/H_2_O to be AIE active. In contrast, reference C2 presented no obvious change in emission as the THF/H_2_O ratio was changed (Fig. 3(b)). The results further indicated that intramolecular H-bonding further rigidified the molecular structure and activated the restriction intramolecular rotation (RIR) process except for intermolecular π–π interactions.

**Fig. 4:**
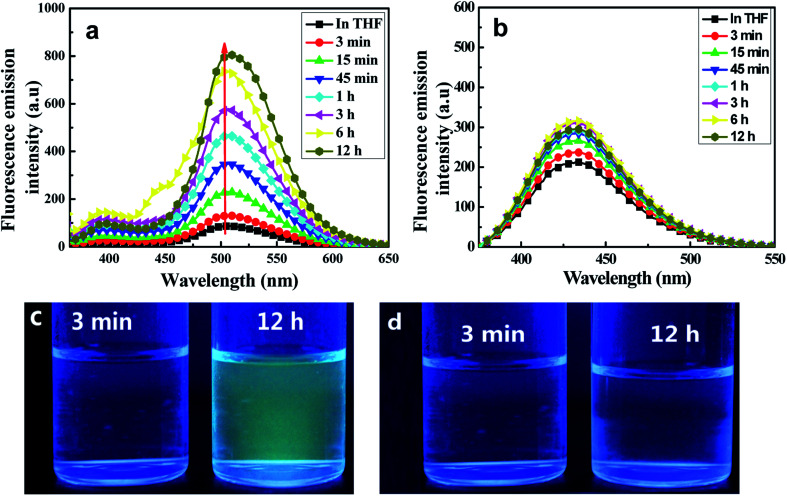
Emission spectra of C1 (a) and C2 (b), each at a concentration of 2 × 10^−5^ mol L^−1^ in a mixed THF/H_2_O (40/60, v/v) solution, at various time points. The excitation wavelength used was 350 nm. Photographs at the indicated time intervals of C1 (c) and C2 (d) under UV lamp irradiation.

Fluorescence emission spectroscopic measurements in THF/H_2_O (40/60, v/v) were taken at various aggregation times. As shown in Fig. 4(a), the target C1 appeared to show weak fluorescence emission in pure THF, whereas the emission of its aggregates in a (40/60, v/v) THF/H_2_O mixed solution was dramatically stronger. The fluorescence emission bands assigned to both enol and keto emissions gradually increased in intensity during the first 45 minutes (*Φ*, 0.158, 45 min), but with a somewhat quicker increase for the keto emission than for the enol emission. Interestingly, the keto emission showed a persistently remarkable increase in intensity after 45 minutes and until 12 hours, after which the intensity remained unchanged. Meanwhile, it displayed an obvious red-shift (7 nm) compared with that in the pure THF solution. In contrast, the intensity of the enol emission hardly changed after 45 minutes. Thus, an intense yellow-green fluorescence emission was observed under UV lamp irradiation (Fig. 4(c)).

The remarkably intensified emission and red-shift of the emission and absorption of C1 in H_2_O/THF (60/40, v/v) compared with that in pure THF further indicated the formation of J-type aggregates (head-to-tail arrangement).^15^ The large Stokes shift (165 nm) of C1 exhibited in H_2_O/THF (60/40, v/v) indicated that the ESIPT process can smoothly proceed in the aggregated state due to the lack in this state of polar solvents disrupting intramolecular hydrogen bonds. At the same time, in the control experiment, the reference molecule without any ESIPT segment could not form aggregates in the THF/H_2_O mixture and the emission intensity showed almost no change (Fig. 4(c)). Furthermore, infrared (IR) spectra of C1 samples in pure THF solution, solid state, and aggregate state, respectively, were obtained (Fig. S5, ESI†). The hydroxyl peak of C1 at about 3439 cm^−1^ was observed here to be much wider than that of the free hydroxyl group. Meanwhile, the positions of the –OH peak in the THF solution (0.1 mol L^−1^), solid state and aggregate state spectra were nearly the same. Therefore, intramolecular H-bonding rather than intermolecular H-bonding was concluded to be present in C1 both in solution and the aggregate state; this result further indicated the important role played by intramolecular H-bonding in the formation of aggregates and emission enhancement of C1 except for intermolecular π–π interaction,^24–26^ which led to the RIR process and rigidified the non-planar configuration in the J-type aggregation state (Fig. S6, ESI†).

In fact, a bright yellow-green emission of C1 was evidently observed in the solid state under UV lamp irradiation (Fig. S7, ESI†). While the reference dye C2 did not display brilliant emission in solid state, in accordance with results discussed above.

## Experimental

**Scheme 1:**
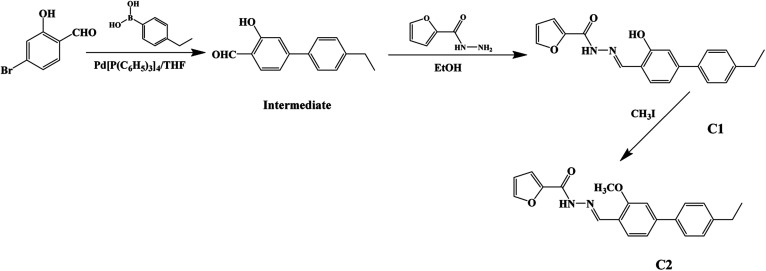
Synthetic routes to the target C1 and reference C2.

**Scheme 2:**
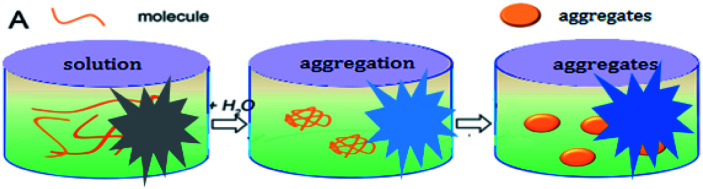
A simulation of the growth of nano-aggregates.

The synthetic route to dyes C1 and C2 is shown in Scheme 1. The details of the synthesis process are shown in ESI.† Nano-aggregates were prepared by rapidly injecting 50 μL of THF solutions of C1 and C2 (2 × 10^−3^ mol L^−1^), respectively, into 50 mL samples of a THF/H_2_O mixed solution (with H_2_O/THF (v/v) ratios from 10% to 90%, respectively) and then vigorously stirring the resulting mixtures at room temperature. When water was appropriately added to the mixed system, the polarity of the mixed system changed dramatically and the free molecules began to aggregate, which inhibited the intramolecular free rotation and non-radiative deactivation channels to some extent through intramolecular H-bonding or π–π interactions. Thus, the fluorescence emission was dramatically enhanced. A simulation of the growth of the nano-aggregates is shown in Scheme 2.

A Bruker 400 MHz NMR instrument was used to record ^1^H and ^13^C nuclear magnetic resonance (NMR) spectra of the samples. A Fourier transform infrared spectrometer was used to acquire IR spectra of the target molecules. Here, an aqueous dispersion of sample dye aggregates in a THF/H_2_O (40/60, v/v) mixture was titrated gradually upon the surface of a plate to prepare predetermined samples of dye aggregates, the solvents were evaporated rapidly, and then the IR spectra of the target dyes were acquired. A TU1901 spectrophotometer from Beijing PUXI General Equipment Limited Corporation was used to acquire UV-visible absorption spectra. The fluorescence spectra were acquired by using a Shimadzu RF-531PC spectrofluorophotometer. The surface morphologies of the aggregates were visualized using a scanning electron microscope from Jeol-JSM-3.5 CF-Japan. Dynamic light scattering (DLS) experiments were performed using an He–Ne laser light source (*λ*, 633 nm), photomultiplier detector, and digital correlator (Malvern Instruments, UK).

## Conclusions

Overall, the dramatic emission enhancement of C1 was achieved through an aggregation process in a mixed solution. By comparing results for C1 and C2, intramolecular H-bonding was concluded to have promoted the aggregation of C1 in the THF/H_2_O mixed solution, and to have hence further enhanced the intensity of the keto peak emission during a certain part of the aggregation time course. As a consequence, a remarkable change from a lack of fluorescence emission to emission of yellow-green light under UV lamp irradiation was observed, which may have strong potential applications in chemical sensing, cell imaging, and so on.

## Conflicts of interest

There are no conflicts to declare.

## Supplementary Material

RA-009-C9RA07290J-s001
