# Supplementary material for: Nano-aggregates of furan-2-carbohydrazide derivatives displaying enhanced emission with a bathochromic shift
Source: RSC Adv. 2019 Nov 6;9(62):36097–102. doi: 10.1039/c9ra07290j (PMC9074951; doi:10.1039/c9ra07290j)

Electronic Supplementary Material (ESI) for RSC Advances.
This journal is © The Royal Society of Chemistry 2019

Supporting Information (SI)

Nano-aggregates of Furan-2-Carbohydrazide derivatives displaying enhanced emission with bathochromic-shift

Ge Ding[*1#], Xinchao Wang[*2#], Xiujuan Li[2], Hongpan Liu[1], Lunxiang Wang[1], Na Liu[1], Fang Gao[*3], Zhenqiang Wang[*4]

[1]College of Materials and Chemical Engineering, Chongqing University of Arts and Sciences, Chongqing, China, 402160

[2]College of Pharmacy, Heze University, Heze, Shandong Province, China, 274000

[3]College of Chemistry and Chemical Engineering, Chongqing University, Chongqing, China, 400044

[4]College of Chemistry, Chongqing Normal University, Chongqing, China, 401331

Emails: dingge1989cqu@126.com, wxc198566@126.com, 772630581@qq.com, fanggao1971@126.com

[#] Xinchao Wang and Ge Ding are designated as the co-first authors, who make equal contribution to this study

Table of contents

Items **pages**

1. Figures of photophysical properties Page 2-4
2. Detail synthesis and characterization Page 5-6
3. ^{1}H NMR spectra of precursor compounds Page 7
4. ^{1}H NMR and ^{13}C-NMR spectra of the target compounds page 8-10

1. Figures of photophysical properties

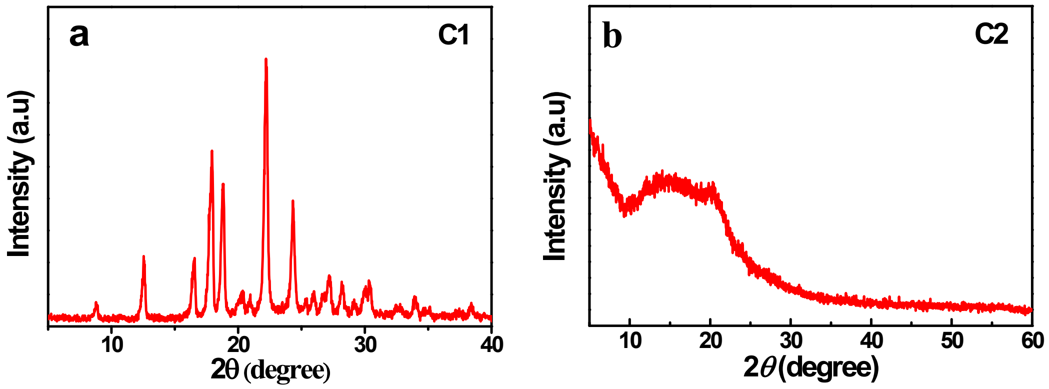

Figure S1 Measured XRD pattern of powder of **C1** (a) and **C2** (b)

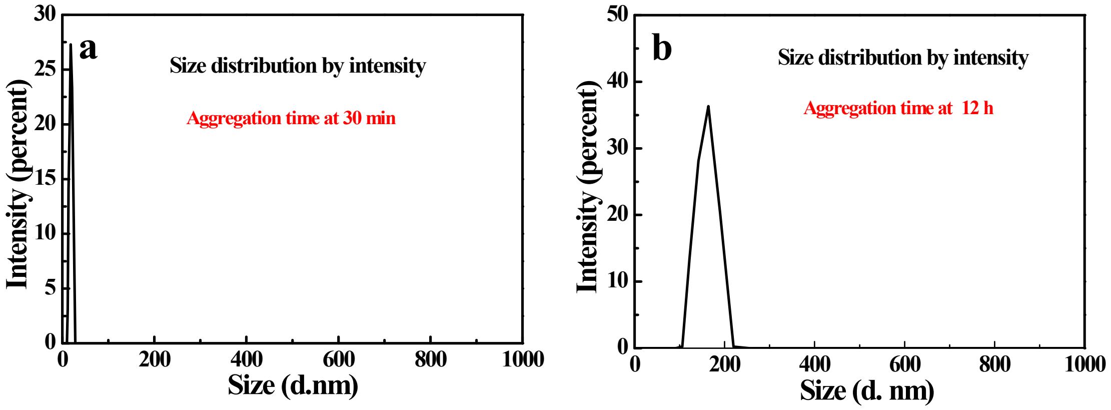

Figure S2 Size distribution of **C1** aggregates (at 30 min (a) and 12 h (b) respectively) in THF/H_2O (40/60 (v/v)) mixture solution (1×10^{-5} mol/L) obtained by DLS.

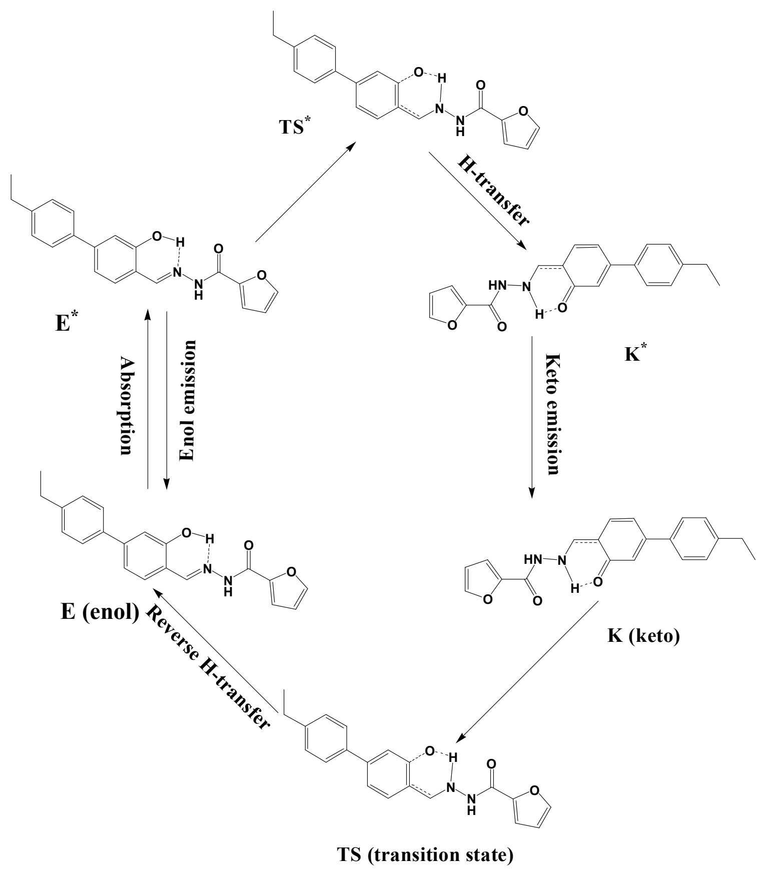

Figure S3 Four-level cycle of internal proton transfer in the excited state for **C1**

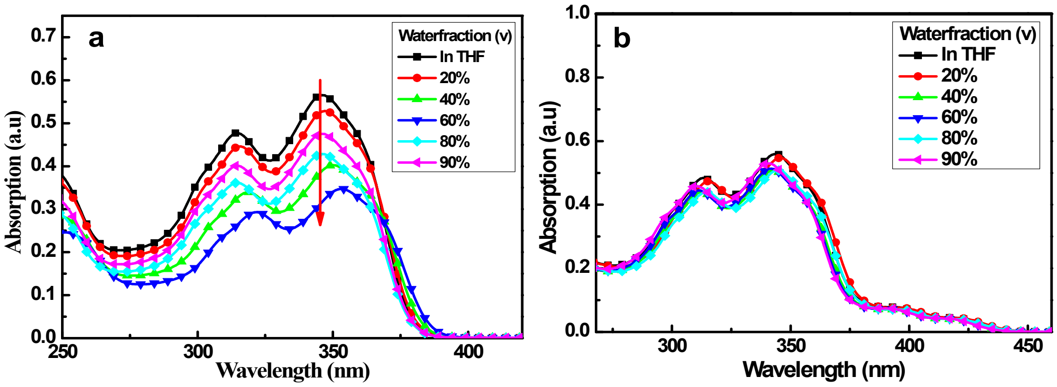

Figure S4 UV/visible absorption spectra of **C1** (a) and **C2** (b) in THF/H_2O mixed solution with different water volume fractions (v), the concentration is 2×10^{-5} mol/L respectively.

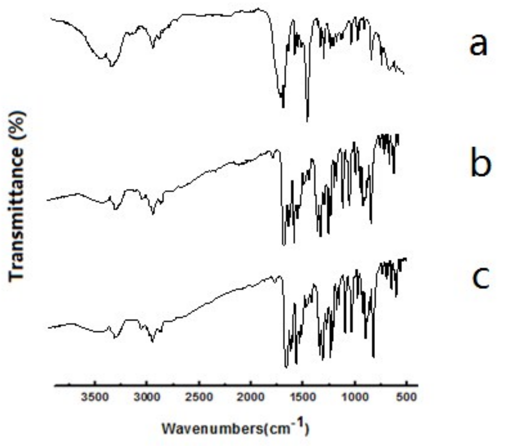

Figure S5 IR spectra of **C1**, (a) in pure THF solution (the concentration is 0.1 mol/L), (b) in solid state and (c) in aggregate state.

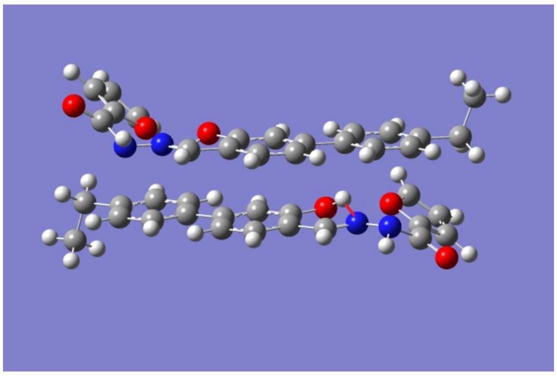

Figure S6 Simulated *J*-type stacking modes of **C1** molecule utilizing the π-π stacking interaction and intramolecular hydrogen bond interaction (O-H···N, red line).

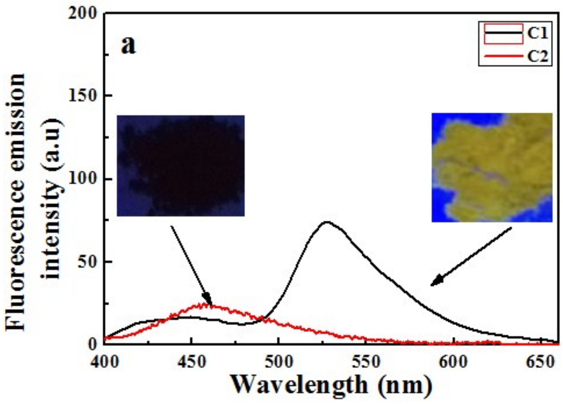

Figure S7 Emission spectra of **C1** and **C2** in solid state, excited at 350 nm, the images are solid state **C1** and **C2** respectively under UV lamp at 365 nm.

2. Detail synthesis and characterization

Intermediate 1

4'-ethyl-3-hydroxy-[1, 1'-biphenyl]-4-carbaldehyde

4-Bromo-2-hydroxybenzaldehyde (1.05 g, 5 mmol) and 4-Ethylphenylboronic acid (2.440 g, 20 mmol) in 100 ml dry THF were added into a three-necked flask. The reaction is carried out under the protection of argon, and potassium carbonate (2.15 g, 15 mmol) was added into the mixed solution after 0.5 h, after 0.5 h of continous reaction, catalyst tetrakis(triphenylphosphine)palladium (112 mg, 0.50 mmol, 5 mol%) was added and reflux for 12 h. After the reaction, the solvent was evaporated under vacuum. Recrystallization from ethanol and cyclohexane gave the pure material as faint yellow solid (yield ~51%, m.p. 210.6～211.8 °C). ^{1}H-NMR (400 MHz, $CDCl_3$) δ (ppm): 11.116 (s, OH, 1H), 9.909 (s, -CH=N-, 1H), 7.609-7.551 (m, Ar-H, 4H), 7.317-7.297 (d, J=8.0 Hz, Ar-H, 2H), 7.243-7.212 (t, J=6.2 Hz, Ar-H, 1H), 2.740-2.633 (t, $-CH_2$, 2H), 1.302-1.264 (t, J=7.6 Hz, $-CH_3$, 3H). Elementary analysis, Anal. Calcd for $C_{15}H_{14}O_2$: C, 79.62; H, 6.24. Found: C, 79.45; H, 6.33.

Target C1

N'-((4'-ethyl-3-hydroxy-[1,1'-biphenyl]-4-yl)methylene)furan-2-carbohydrazide

4'-ethyl-3-hydroxy-[1,1'-biphenyl]-4-carbaldehyde (1.05 g, 5 mmol) and Furan-2-Carbohydrazide (2.440 g, 20 mmol) in 100 ml dry ethanol were added into a three-necked flask. The reaction mixture was stirred at room temperature under argon protection for 12 hours. After the reaction, the solvent was evaporated under vacuum. Recrystallization from ethanol and cyclohexane gave the pure material as faint yellow solid (yield ~62%, m.p. 256.6～257.8 °C). ^{1}H-NMR (400 MHz, DMSO-d_6) δ (ppm): 12.123 (s, -OH, 1H), 11.206 (s, -NH-, 1H), 8.619 (s, -

CH=N-, 1H), 7.909-7.870 (m, Ar-H, 1H), 7.621-7.513 (m, furan-H, 3H) , 7.294-7.274 (d, *J*=8.0 Hz, Ar-H, 2H), 7.208-7.128 (m, Ar-H, 4H), 2.646-2.590 (m, $-CH_2-$, 2H), 1.200-1.162 (t, *J*=16 Hz, $-CH_3$, 3H). ^{13}C-NMR (151 MHz, DMSO-d_6) δ (ppm): 168.263, 163.287, 145.110, 140.120, 139.264, 138.336, 138.115, 137.582, 132.605, 129.608, 128.860, 127.046, 126.604, 118.182, 113.876, 111.594, 106.739, 106.704, 28.261, 15.992. Elementary analysis, Anal. Calcd for $C_{20}H_{18}N_2O_3$: C, 71.84; H, 5.43; N, 8.38. Found: C, 71.92; H, 5.31; N, 8.32.

Reference C2

N'-((4'-ethyl-3-methoxy-[1,1'-biphenyl]-4-yl)methylene)furan-2-carbohydrazide

N'-((4'-ethyl-3-hydroxy-[1,1'-biphenyl]-4-yl)methylene)furan-2-carbohydrazide (0.94 g, 10 mmol) and sodium hydroxide (1.60 g, 40 mmol) in DMSO (20 mL) was treated with methyl iodide (2.48 g, 20 mmol) and maintained at room temperature for 30 min. The reaction mixture was diluted with water (20 mL) and extracted with light petroleum. The combined petroleum extracts were washed with aqueous sodium hydroxide and dried with $MgSO_4$, the solvent was evaporated under vacuum. Recrystallization from ethanol and cyclohexane gave the pure material as faint yellow solid (yield ~66%, m.p. 260.6～261.9 °C). ^{1}H-NMR (400 MHz, DMSO-d_6) δ (ppm):11.207 (s, -NH-, 1H), 8.619 (s, CH=N-, 1H), 7.939-7.867 (m, Ar-H, 3H), 7.617-7.491 (m, furan-H, 3H), 7.239-7.128 (m, Ar-H, 5H), 3.757 (s, $-OCH_3$, 3H), 3.354-3.329 (m, $-CH_2-$, 2H), 1.089-1.086 (d, *J*=12 Hz, $-CH_3$). ^{13}C-NMR (151 MHz, DMSO-d_6) δ (ppm): 168.672, 145.738, 144.452, 141.957, 141.364, 138.734, 137.254, 128.423, 127.060, 126.520, 123.775, 123.733, 122.070, 115.343, 56.981, 29.736, 15.515. Elementary analysis, Anal. Calcd for $C_{21}H_{20}N_2O_3$: C, 72.40; H, 5.79; N, 8.04. Found: C, 72.38; H, 5.75; N, 8.21.

3. ^{1}H NMR spectra of precursor compounds

Intermediate

4'-ethyl-3-hydroxy-[1,1'-biphenyl]-4-carbaldehyde

^{1}H-NMR ($CDCl_3$)

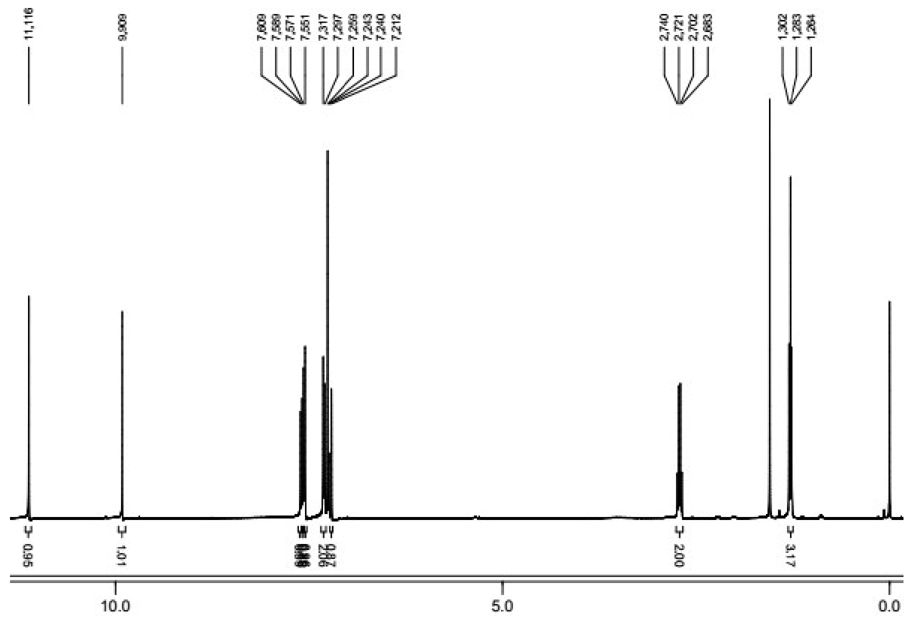

4. The characterization of the target compounds

Target molecule C1

^{1}H-NMR (DMSO-d_6)

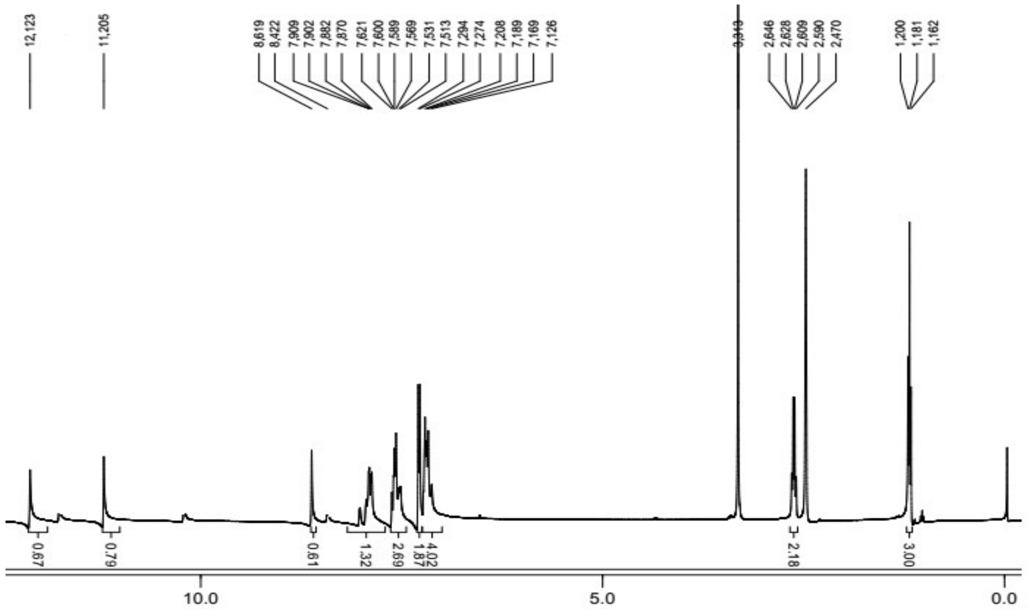

^{13}C-NMR (DMSO-d_6)

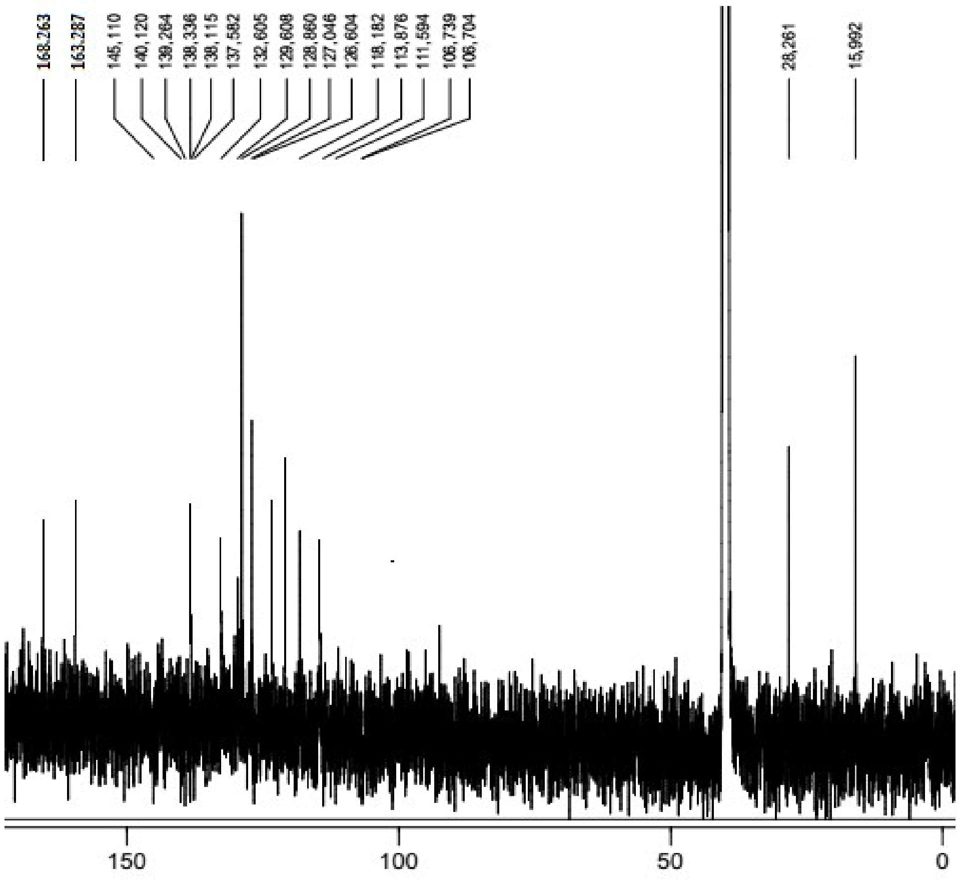

***Reference* C2**

^{1}H-NMR (DMSO-d_6)

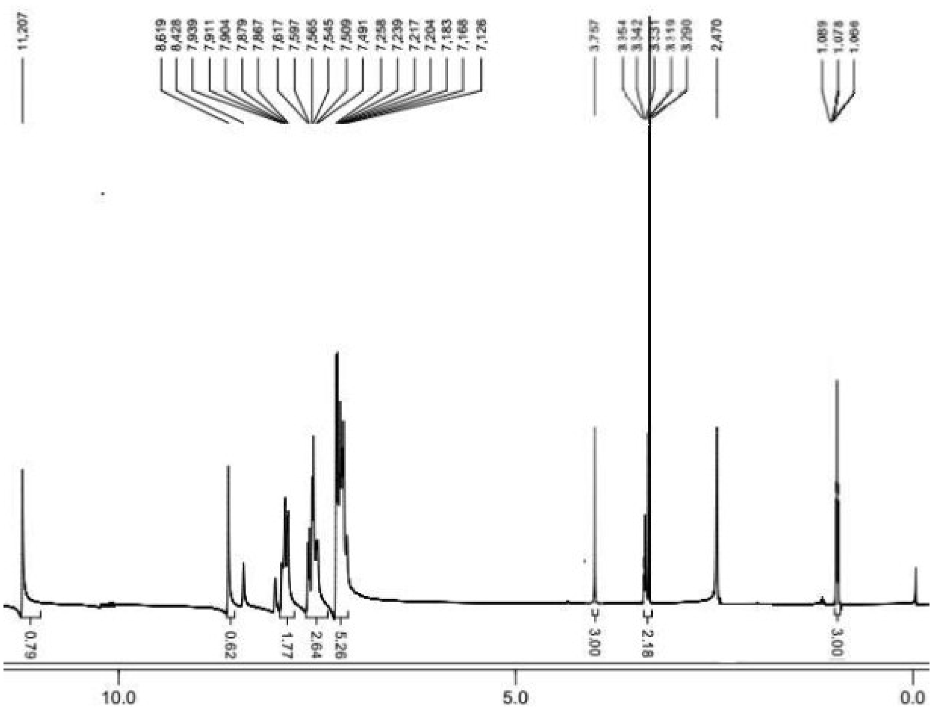

^{13}C-NMR (DMSO-d_6)

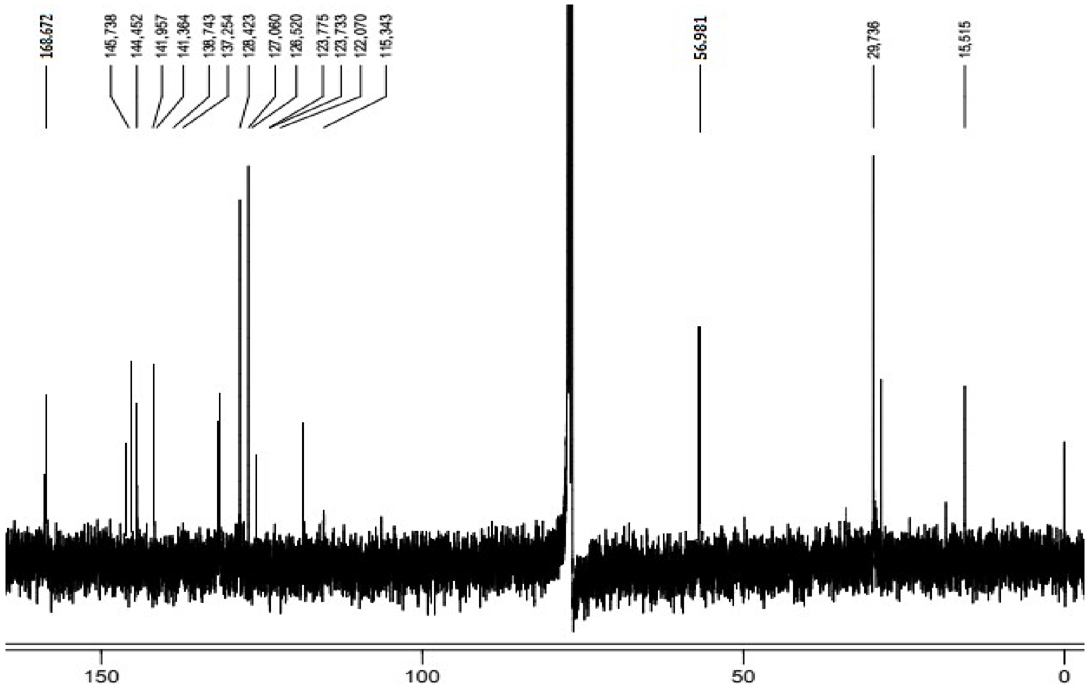

Supplement: RA-009-C9RA07290J-s001 [file RA-009-C9RA07290J-s001.pdf]
